# Progenitor Cell Dynamics in Androgenetic Alopecia: Insights from Spatially Resolved Transcriptomics

**DOI:** 10.3390/ijms26125792

**Published:** 2025-06-17

**Authors:** Sasin Charoensuksira, Piyaporn Surinlert, Aungkana Krajarng, Thararat Nualsanit, Witchuda Payuhakrit, Pimchanok Panpinyaporn, Wilunplus Khumsri, Wilai Thanasarnaksorn, Atchima Suwanchinda, Suradej Hongeng, Saranyoo Ponnikorn

**Affiliations:** 1 Division of Dermatology, Chulabhorn International College of Medicine, Thammasat University, Pathum Thani 12120, Thailand; sasinboong@gmail.com (S.C.);; 2 Chulabhorn International College of Medicine, Thammasat University, Pathum Thani 12120, Thailand; 3 Research Unit in Synthesis and Applications of Graphene, Thammasat University, Pathum Thani 12120, Thailand; 4 Department of Pathobiology, Faculty of Science, Mahidol University, Bangkok 10400, Thailand; 5 Pathobiology Information and Learning Center, Department of Pathobiology, Faculty of Science, Mahidol University, Bangkok 10400, Thailand; 6 Interdisciplinary Program of Biomedical Sciences, Graduate School, Chulalongkorn University, Bangkok 10330, Thailand; 7 Division of Dermatology, Faculty of Medicine, Ramathibodi Hospital, Mahidol University, Bangkok 10400, Thailand; 8 Division of Hematology and Oncology, Department of Pediatrics, Faculty of Medicine, Ramathibodi Hospital, Mahidol University, Bangkok 10400, Thailand; 9 Thammasat University, Pattaya Campus, Bang Lamung 20150, Thailand

**Keywords:** androgenetic alopecia, spatial transcriptomics, epithelial–mesenchymal transition, extracellular matrix, progenitor cell loss

## Abstract

Androgenetic alopecia (AGA) is marked by the progressive miniaturization of hair follicles (HFs) and hair thinning, driven by a decline in the progenitor cells critical for hair regeneration. Despite this, the mechanisms responsible for progenitor cell depletion remain largely unclear. To investigate transcriptional alterations in the progenitor cell regions of AGA patients while maintaining the spatial tissue context, we employed the GeoMX Digital Spatial Profiling (DSP) platform, which enables a precise comparison with healthy controls. Our analysis revealed the significant upregulation of genes associated with extracellular matrix (ECM) organization and the epithelial–mesenchymal transition (EMT), including *FN1*, *TWIST1*, and *TGFB2* in the progenitor cell region of the HFs. Correspondingly, protein expression data confirmed increased levels of the protein products of these genes in the affected areas, underscoring their roles in the disease’s progression. These molecular changes suggest an environment conducive to the EMT, potentially contributing to the loss of progenitor cells and indicating a fibrogenic shift within the HF microenvironment. Additionally, our study highlights the influence of peri-infundibular immune cell infiltration on these molecular changes, suggesting that immune-mediated microinflammation may contribute to the fibrogenic environment and progenitor cell loss in the AGA. These findings demonstrate the utility of spatial transcriptomics in identifying potential therapeutic targets and advancing our understanding of AGA’s molecular mechanisms, offering avenues for developing targeted treatment strategies.

## 1. Introduction

Androgenetic alopecia (AGA) is men’s most prevalent form of hair loss, characterized by a genetic predisposition and increased sensitivity to androgens [1,2,3]. The condition is marked by hair follicle (HF) miniaturization and alterations in the hair growth cycle, including a shortened growth phase and an extended resting phase [4,5,6]. The dysregulation of factors secreted by dermal papilla (DP) cells, crucial for hair cycle regulation, contributes to these changes [7,8,9,10,11,12,13].

The HF is a complex structure comprising an epithelial compartment that includes HF stem cells (HFSCs) and their progeny. These cells interact with the surrounding microenvironment, including the DP, playing a pivotal role in regulating hair growth [14]. A significant aspect of AGA pathology involves the depletion of the CD34+ progenitor cells necessary for generating the lower HF epithelium [15]. This population decreases dramatically and earlier than the CD200+ HFSC population during miniaturization [16,17], suggesting a significant contribution to the process. Additionally, the role of extra-bulge epithelial cells in regenerating the lower segment of anagen HFs following mild injury highlights the importance of progenitor cells in preventing a premature transition to the catagen phase [18]. There is a hypothesis that the loss of CD34+ progenitor cells is possibly due to the failure of conversion from bulge HFSCs. However, the exact and comprehensive mechanism remains unclear. Here, we propose that the decrease in progenitor cells in AGA is not solely due to a conversion defect from bulge HFSCs, but may also stem from molecular disturbances within the progenitor cells.

Advances in transcriptomics, particularly single-cell RNA sequencing (scRNA-seq) and spatial transcriptomics, have transformed our understanding of complex tissues by enabling high-resolution, cell-type-specific gene expression profiling [19,20,21]. While previous studies of AGA have utilized bulk RNA sequencing to compare affected and unaffected HFs, such approaches lack the resolution needed to distinguish between specific cellular populations within the HF niche. A recent study that employed location-specific transcriptome profiling of HFs from AGA patients versus control donors has begun to address this limitation by micro-dissecting the bulge-containing and DP-containing portions [22]. However, techniques capable of dissecting tissue heterogeneity with a higher resolution are needed to capture more precise changes among heterogeneous cell types.

Single-cell RNA sequencing (scRNA-seq) has emerged as a powerful technique for revealing cellular heterogeneity and the unique molecular signatures of each cell type within the HFs [23,24,25,26]. However, scRNA-seq requires cell dissociation from their native context, resulting in the loss of spatial information. Although recent computational methods can partially infer spatial information by referencing existing spatial atlases [27], these inferences are limited by the accuracy and completeness of the reference datasets. In contrast to prior approaches, spatial transcriptomics technologies such as GeoMX Digital Spatial Profiling (DSP) preserve the architectural organization of tissues, enabling the direct, high-resolution measurement of gene expression within defined anatomical regions. By leveraging this approach, we profiled the transcriptional signature of progenitor cell regions in AGA patients (PG-A) and in analogous areas from control donors (PG-C), preserving the tissue architecture of the scalp. This allowed us to uncover the associated candidate pathways and biological processes involved in hair progenitor cell loss from the HF niche. To our knowledge, this study provides the first spatially resolved atlas of gene expression perturbations in HF progenitor cells from patients with androgenetic alopecia (AGA), offering novel mechanistic insights and a reference resource not achievable with previous techniques.

## 2. Results

### 2.1. PG-A and PG-C Exhibit Distinct Transcriptomic Profiles

**Figure 1:**
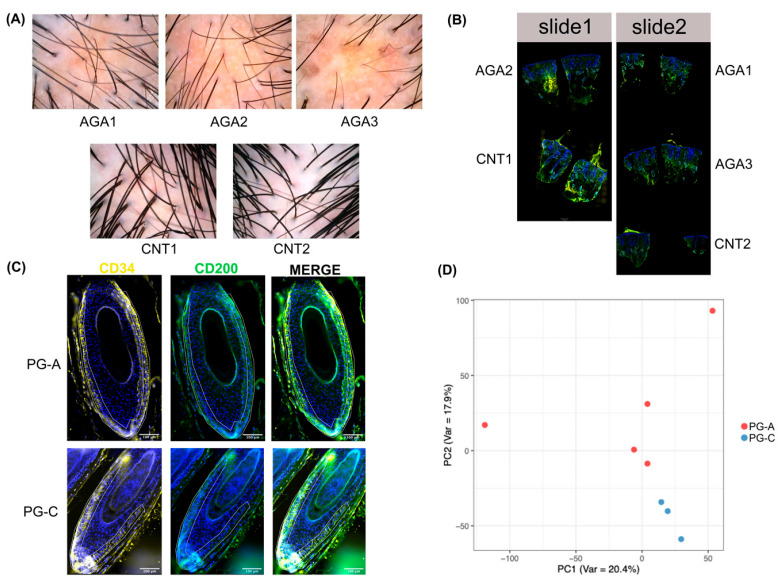
(**A**) Dermoscopic images of androgenetic alopecia patients (AGA1, AGA2, and AGA3) alongside control donors (CNT1 and CNT2). (**B**) Distribution of scalp tissue sections across a total of two slides. (**C**) Representative immunofluorescent staining depicting CD34 (yellow), CD200 (green), and nuclear staining with Syto83 (blue). Scale bars represent 100 µm. White polygonal outlines denote manually delineated boundaries for region-of-interest (ROI) selection. (**D**) Principal component analysis (PCA) of the transcriptional profiles for all ROIs.

To investigate the transcriptomic differences in progenitor cell regions between AGA patients and control donors, we analyzed scalp tissue biopsies from three clinically and dermoscopically diagnosed AGA patients and two control donors, as shown in Figure 1A and detailed in Table S1. Utilizing the GeoMX DSP platform in conjunction with the Whole Transcriptome Atlas (WTA), we assessed 18,318 human transcripts. Tissue sections from these biopsies were arranged on two slides (Figure 1B). Immunofluorescence staining was employed to identify the HF progenitor cells, characterized as basal keratinocytes in the lower segment of the HFs, which were strongly positive for CD34 and either negative or weakly positive for CD200, as depicted in Figure 1C. We then selected progenitor cell areas as ROIs for transcriptional profiling, designating one ROI per HF. In total, eight ROIs were delineated, with five from the AGA group (PG-A) and three from the control group (PG-C).

To validate the purity of the selected population, the ROI in the upper HF segment (UHF), which contains both the HF isthmus and the sebaceous gland area, was used as an internal control to confirm the enrichment of CD34^+^ cells in the PG-A and PG-C ROIs (Figure S1A). Since progenitor cells are CD34^+^CD200^−^ basal keratinocytes with a high proliferative capacity, the expression levels of *CD34* (a progenitor cell marker), *CD200* (a HFSC marker) (25), integrin subunit beta 1 (*ITGB1*, a basal cell marker) (38), marker of proliferation Ki-67 (*MKI67*, a proliferation marker) (39), and cyclin D1 (*CCND1*, a cell cycle progression marker) (40), were compared between the PG and the UHF. The comparison revealed the expected higher expression of *CD34*, *ITGB1*, and *CCDN1*, but a lower expression of *CD200*, in the PG area (Supplementary Figure S1B), indicating that progenitor cells were enriched in this area. Additionally, no significant differences were observed in the expression of *CD34*, *ITGB1*, *MKI67*, *CCDN1*, and keratin 10 (*KRT10*, a keratinocyte differentiation marker) (41) between PG-A and PG-C, suggesting that both are similar cell populations (Supplementary Figure S1C).

The transcriptomic profiles of all the regions of interest (ROIs) were compared. The principal component analysis (PCA) of the ROIs revealed a separation between the gene expression profiles of PG-A and PG-C (Figure 1D), indicating a transcriptional distinction in HF progenitor cells between these two groups.

### 2.2. Transcriptional Signatures and Pathway Enrichment in HF Progenitor Cells Indicate Predilection for Epithelial–Mesenchymal Transition (EMT)

**Figure 2:**
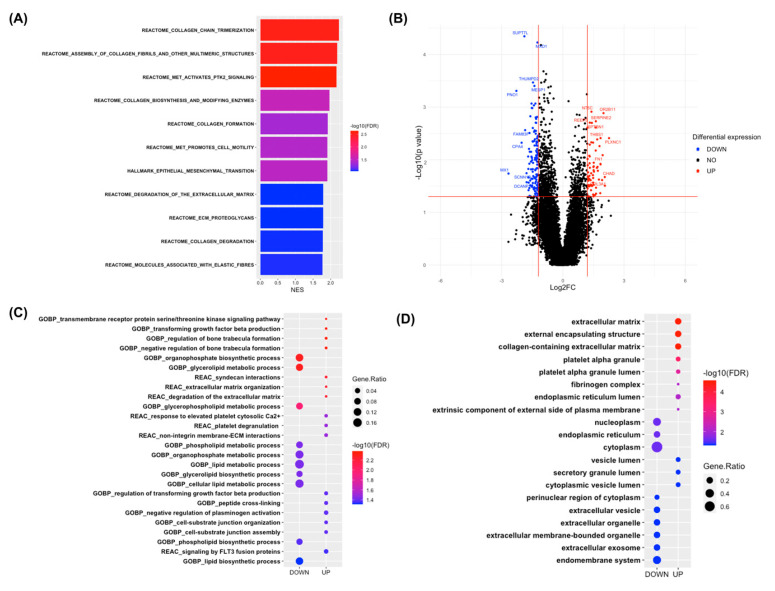
(**A**) Bar chart showing significantly enriched gene sets in PG-A versus PG-C with FDR < 0.1, using a GSEA with the Hallmark and Reactome gene sets. The *y*-axis lists the gene sets, and the *x*-axis shows their normalized enrichment scores (NES). Bar colors correspond to the −log10(FDR) values. (**B**) Volcano plot displaying differentially expressed genes between PG-A and PG-C, with red indicating upregulation, blue indicating downregulation, and black showing no significant change. FC denotes fold change. (**C**,**D**) Dot plots for the overrepresentation analysis of DEGs. (**C**) shows the GOBP terms and Reactome pathways with FDR < 0.05 for upregulated or downregulated DEGs. (**D**) shows the GOCC terms with FDR < 0.05 for DEGs. The *y*-axis lists significant terms, the dot size represents the gene ratio, and the dot color reflects −log10(FDR) from Fisher’s exact test.

To gain insights into the biological processes disturbed in PG-A compared with PG-C, we conducted a gene set enrichment analysis (GSEA) to identify potential pathways in which several genes each change a small amount, but in a coordinated way (42). The GSEA was performed on the expression dataset against the Reactome and Hallmark gene sets. Several biological processes, including “collagen chain trimerization”, “collagen biosynthesis and modifying enzymes”, “collagen formation”, “epithelial–mesenchymal transition”, etc., were identified to be enriched in PG-A (Figure 2A and Supplementary Table S3).

Upon a differential expression analysis, 197 differentially expressed genes (DEGs) were identified (66 upregulated and 131 downregulated) (Figure 2B and Supplementary Table S2). To understand the altered pathways and biological processes in PG-A versus PG-C based on these differentially expressed genes (DEGs), the g: Profiler server was used for a functional and pathway enrichment analysis of the DEGs against the Gene Ontology biological process (GOBP), Reactome, and Gene Ontology cellular component (GOCC) categories. A Benjamini-Hochberg FDR of less than 0.05 was set for statistical significance. The bubble plot demonstrates that upregulated genes are mainly involved in extracellular matrix organization, extracellular matrix degradation, and transforming growth factor beta production, as shown in Figure 2C and Supplementary Table S4. The upregulated genes are present in the extracellular matrix, the external encapsulating structure, and the collagen-containing extracellular matrix, as shown in Figure 2D and Supplementary Table S5. The downregulated genes are present in various cellular compartments, including the cytoplasm, endomembrane system, and nucleoplasm, as shown in the GOCC section of the ontology terms (Figure 2D and Supplementary Table S5). Additionally, the downregulated genes are involved in lipid metabolism processes, lipid biosynthesis processes, and organophosphate metabolic processes, among others (Figure 2C and Supplementary Table S4).

**Figure 3:**
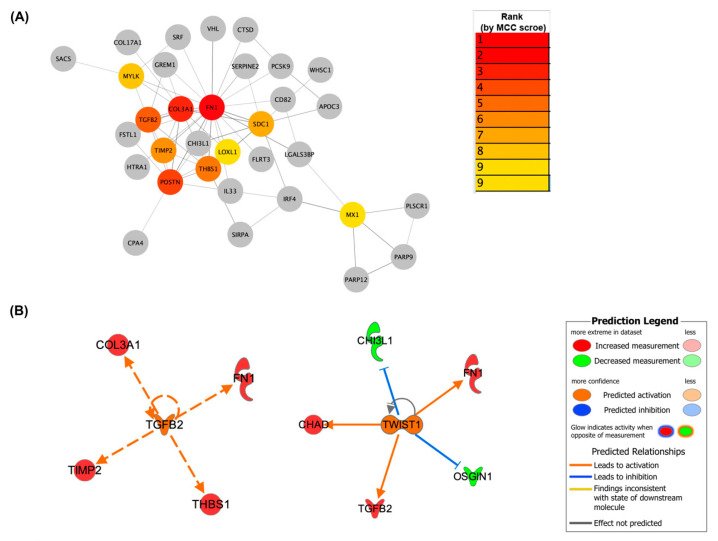
(**A**) The top 10 hub genes, ranked by the MCC algorithm and their neighbors among all the DEGs, using the CytoHubba Cytoscape plugin. The connection degree level of hubs is represented by a color scale from red (highest degree) to yellow (lower degree). (**B**) An upstream regulator IPA. TGFB2 and TWIST1 are highlighted in orange as the top-predicted activated upstream regulators, with significantly overlapping p-values and a high directional consistency (z-scores). The network displays upstream regulators and their targets, with the expression trends indicated. Upregulated and downregulated genes are shown in red and green, respectively. Orange and blue arrows represent activation and inhibition, while dashed and solid lines indicate indirect and direct interactions, respectively. The prediction legend key of the IPA is in the right corner.

Moreover, the protein–protein interaction (PPI) network was constructed using the STRING app version 2.0.1 in Cytoscape software version 3.9.1. To identify high-degree genes that play a critical role in the PPI, the CytoHubba version 1.5.1, a plugin in Cytoscape, was used. This plugin identified genes with a high degree of centrality. The hub genes were selected from the PPI network using the maximal clique centrality (MCC) algorithm of the CytoHubba plugin. According to the MCC scores, the top ten highest-scored genes, including fibronectin 1 (*FN1*), collagen type III alpha 1 chain (*COL3A1*), periostin (*POSTN*), transforming growth factor beta 2 (*TGFB2*), thrombospondin 1 (*THBS1*), tissue inhibitor of metalloproteinases 2 (*TIMP2*), syndecan 1 (*SDC1*), myosin light chain kinase (*MYLK*), lysyl oxidase-like 1 (*LOXL1*), and myxovirus resistance 1 (*MX1*), were selected as the hub genes (Figure 3A). Among these hub genes, *FN1*, *COL3A1*, *POSTN*, *TGFB2*, *THBS1*, and *TIMP2* were upregulated, while *SDC1*, *MYLK*, *LOXL1*, and *MX1* were downregulated. Notably, *FN1* had the highest MCC score, suggesting that it is the most important gene in the network. A pathway enrichment analysis of these ten hub genes showed that they were primarily enriched in “extracellular matrix organization” according to the Reactome database (FDR = 4.41 × 10^−8^).

We used Ingenuity Pathway Analysis (IPA) software (version 22.0) to identify the upstream regulators driving the DEGs. Among the predicted upstream regulators with an overlap *p*-value < 0.05 and an absolute z-score > 2, *TWIST1* (*p*-value of overlap = 0.0416, activation z-score = 2.164) and TGFB2 (*p*-value of overlap = 0.00894, activation z-score = 2.118) were predicted to be activated upstream regulators (Figure 3B). *TGFB2* is also an upregulated hub gene identified in PPI. These two predicted upstream regulators play a critical role in fibrosis and the EMT (43, 44). *TGFB2* was predicted to activate a network of fibrosis- and ECM-related genes, including *FN1*, *TIMP2*, *THBS1*, and *COL3A1*. *TWIST1* was predicted to activate FN1, TGFB2, and chondroadherin (*CHAD*) while inhibiting oxidative stress-induced growth inhibitor 1 (*OSGIN1*) and chitinase 3-like 1 (*CHI3L1*).

**Figure 4:**
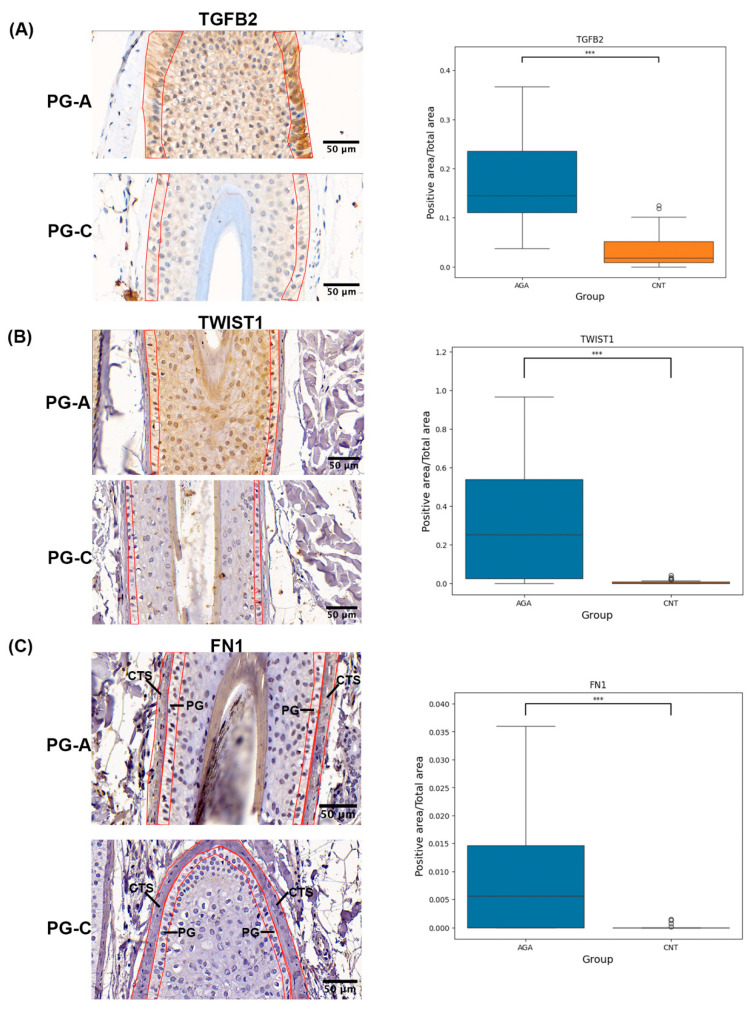
Comparative analysis of FN1, TWIST1, and TGFβ2 protein expression and localization in the PG-A and PG-C groups. The left panels display IHC staining for each protein. Red outlines indicate the regions of interest (ROIs) that were selected for quantitative analysis. The right panels show box plots of the positive fractional area, indicating the level of protein expression, with the *y*-axis representing the positive fractional area and the *x*-axis differentiating between the PG-A and PG-C groups. (**A**) The TGFβ2 expression was significantly higher in the PG-A group compared to PG-C. (**B**) TWIST1 showed an increased expression in PG-A. (**C**) The FN1 expression was significantly higher in the connective tissue sheath (CTS) adjacent to the PG-A group compared to PG-C, with IHC staining in the CTS due to an unobservable signal in the PG area. Asterisks denote statistical significance (*** *p* < 0.001).

All the above results suggest that the cells in PG-A are more likely to undergo an epithelial-to-mesenchymal transition (EMT). To confirm this conclusion, the protein expression of candidate genes involved in the EMT, including *TGFB2*, *TWIST1*, and *FN1*, was characterized using an immunohistochemical (IHC) analysis (Figure 4). The IHC analysis was employed in seven patients with AGA and five control donors, specifically locating the progenitor cells, characterized as basal keratinocytes within the sub-bulge and supra-bulbar areas of the HF. FN1 was barely detectable in the PG and the supra-basal keratinocytes; however, the IHC analysis showed a substantial increase in the FN1-positive area within the connective tissue sheath (CTS) adjacent to the PG-A region. Additionally, areas that stained positive for TWIST1 and TGFβ2 were considerably more significant in PG-A. The notable accumulation of FN1 in the connective tissue adjacent to PG-A, coupled with the increased expression of TWIST1 and TGFβ2, indicates a proclivity for the EMT and fibrotic processes within the PG-A cellular milieu. This environment may contribute to the progression of perifollicular fibrosis and the potential loss of PG, which are characteristic features of AGA.

### 2.3. Influence of the Immune Microenvironment on the EMT and Fibrotic Processes in Progenitor Cells of AGA

Our previous studies have explored the transcriptomic profiles of perifollicular immune cells surrounding the infundibulum (peri-infundibular area), an area often associated with microinflammation in androgenetic alopecia (AGA) [28]. Compelling evidence increasingly supports the notion that persistent inflammation actively contributes to the development of perifollicular fibrosis in the later stages of the disease. We suggest that there may be potential crosstalk between these immune cell clusters and HF progenitor cells.

To investigate this, we utilized immune cell datasets derived from our previously published spatial transcriptomic analysis [28]. In that study, immune cell populations within the peri-infundibular region were characterized using GeoMX DSP. Immune-enriched areas were first identified by selecting regions with a high CD45^+^ cell density—a pan-leukocyte marker. GeoMx DSP’s segmentation capability was then applied to isolate and analyze the gene expression specifically from CD45^+^ immune cells. The DEGs were identified as previously described.

**Figure 5:**
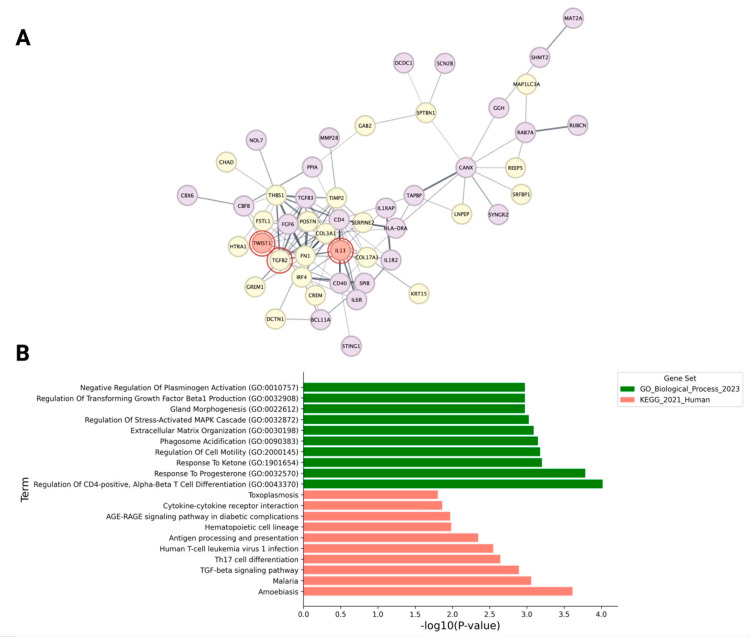
(**A**) Protein–protein interaction (PPI) network between upregulated genes in perifollicular immune cells and hair follicle (HF) progenitor cells in androgenetic alopecia (AGA). Each node represents a gene, and the edges denote functional interactions. Yellow nodes correspond to genes upregulated in the progenitor cell dataset, while purple nodes correspond to genes upregulated in the immune cell dataset. Red nodes with red circles indicate predicted activated upstream regulators identified by IPA that were not detected as differentially expressed genes (DEGs). Among these, *TWIST1* was identified from the progenitor cell dataset, while *IL13* was identified from the immune cell dataset. (**B**) Functional enrichment analysis of upregulated genes from both the progenitor and immune cell datasets. The top ten enriched biological processes (green bars) from the Gene Ontology Biological Process (GOBP) and the top ten pathways (pink bars) from the KEGG pathway analysis are shown. Bars indicate the statistical significance of the enrichment, represented as −log10 (*p*-value) for each term.

We executed another PPI network analysis to probe the potential interactions between immune and progenitor cells in androgenetic alopecia. This network, depicted in Figure 5A, comprises all upregulated differentially expressed genes (DEGs) from immune and progenitor cell datasets, alongside activated upstream regulators predicted by IPA software version 22.0. Among these, *TGFB2* was identified as an upregulated differentially expressed gene (DEG) and an activated upstream regulator within the progenitor dataset. Although not initially identified as differentially expressed genes (DEGs), the regulators *TWIST1* and *IL13* were activated in the progenitor and immune cell datasets, respectively. Their inclusion expanded the PPI network, showing connectivity among these regulators, with key hub genes such as *FN1* and *CD4* serving as vital links between the progenitor and immune compartments.

A subsequent functional enrichment analysis, illustrated in Figure 5B and Supplementary Table S6, targeted biological processes associated with the upregulated genes. This analysis revealed significant enrichment (*p*-value < 0.05) in immune-related processes, including the “regulation of CD4-positive, alpha-beta T cell differentiation”, “cytokine-cytokine receptor interaction”, and “antigen processing and presentation”, underscoring the role of immune modulation in creating a microinflammatory environment surrounding the HF. Furthermore, pathways linked to fibrosis and the EMT, such as “extracellular matrix organization”, the “regulation of transforming growth factor beta1 production”, and the “TGF-β signaling pathway”, were prominently enriched.

Collectively, these findings depict a dynamic, bidirectional interaction between progenitor and immune cells within the HF microenvironment. These interactions are likely instrumental in the observed fibrogenic shift and the EMT in progenitor cells affected by AGA, emphasizing the intricate role of immune modulation in the progression of this disease.

## 3. Discussion

The findings from our study provide significant insights into the complex pathophysiology of AGA, with a primary focus on the molecular alterations in progenitor cells. The use of spatial transcriptomics has been instrumental in unraveling these intricate mechanisms by preserving the spatial context of the tissue, enabling the precise profiling of specific cells at their exact locations. This advanced technique has helped us to dissect the transcriptional changes occurring within the HF progenitor cell regions of AGA patients compared to control donors, shedding light on the molecular underpinnings of AGA.

Previous studies have highlighted the significant depletion of CD34^+^ progenitor cells in AGA, which plays a crucial role in the miniaturization process of HFs [16,17]. This depletion is thought to be attributable to a conversion defect in bulge HFSCs. Here, we added the link to molecular disturbances within the progenitor cells, potentially leading to their loss through the EMT and fibrosis.

The link between perifollicular fibrosis and AGA is well-established, often occurring alongside perifollicular microinflammation [29,30]. This fibrotic development is also driven by androgens, specifically through testosterone, which elevates type I procollagen and TGF-β1 levels in human scalp dermal fibroblasts [12]. Our research builds on this understanding by exploring the role of epithelial progenitor cells in this process. We identified the upregulation of key extracellular matrix-related factors, including *FN1*, *TWIST1*, and *TGFB2*, at both the gene and protein levels in the progenitor cell compartment. Notably, several other hub genes and genes associated with upstream regulators highlighted in our study—such as *TIMP2*, *POSTN*, and *CHI3L1*—are also critical regulators of ECM remodeling in other degenerative and fibrotic conditions, including osteoarthritis [31]. This suggests a heightened likelihood of EMT processes and fibrotic changes within the progenitor cell niche. These molecular pathways are likely active in scalps affected by AGA, contributing to the observed fibrotic alterations in the perifollicular space and the concurrent loss of progenitor cells. This is consistent with findings in HFSCs within miniaturized HFs, which display fibroblast-like characteristics and increased levels of fibrosis markers [32]. These insights suggest that the EMT and fibrosis may impair both epithelial stem cells and progenitor cells, thereby exacerbating perifollicular fibrosis and leading to epithelial cell insufficiency in AGA.

FN1 is crucial in tissue repair, fibrosis, and EMT processes, acting as an essential scaffold that facilitates fibroblast attachment and migration. This is necessary for the deposition of new extracellular matrix components [33,34,35]. Predominantly located in the basement membrane and dermal components of HFs, FN1 supports cellular adhesion and mobility and is vital for remodeling the basement membrane [36,37]. While FN1 is mainly present in these areas, its production is not exclusively dermal; epidermal keratinocytes have also been demonstrated to synthesize FN1 protein [38]. Although an immunohistochemical analysis showed a limited sensitivity in detecting FN1 protein in progenitor cells, there is evidence of increased FN1 gene expression in these cells, with subsequent deposition in the adjacent basement membrane and CTS. These findings suggest increased FN1 synthesis. The significant accumulation of FN1 in the basement membrane and CTS surrounding HFs likely contributes to the fibrotic encapsulation, resulting in a reduction in follicle size and narrower hair shafts, which are characteristic features of AGA. Another hub gene, *POSTN*, which interacts with *FN1* and many other genes, is a matricellular protein that supports collagen crosslinking, fibroblast activation, and TGF-β signaling, all of which contribute to a stiffened fibrotic ECM [39]. Elevated *POSTN* expression has been associated with scarring alopecia [40].

TGF-β2 is recognized for its ability to trigger the catagen phase in HFs. The role of TGF-β2 in initiating the catagen phase is pivotal in the pathogenesis of AGA. Dermal papilla (DP) cells, upon stimulation by dihydrotestosterone (DHT), produce TGF-β2, which triggers a cascade of biochemical events culminating in the apoptotic death of epithelial cells [13]. Beyond its role in epithelial apoptosis, TGF-β2 facilitates the EMT and fibrosis by promoting the synthesis of ECM proteins [41]. In our analysis, *TGFB2* was predicted to activate multiple downstream ECM-related genes, including *FN1*, *TIMP2*, *THBS1*, and *COL3A1*. The function of *TIMP2* is context-dependent, but classically, it limits ECM degradation by inhibiting matrix metalloproteinases, thus promoting matrix accumulation when overexpressed [42]. *COL3A1*, encoding collagen type III, is a major fibrillar component deposited during fibrotic remodeling [43]. *THBS1* is a multifunctional glycoprotein that not only activates latent TGF-β, but also mediates fibroblast recruitment and ECM organization [44,45]. These interactions suggest that TGF-β2 operates as a master regulator of the fibrotic microenvironment in AGA, driving the expression of multiple effectors involved in extracellular matrix stabilization, remodeling, and EMT induction in HF epithelial progenitor cells. Our findings indicate a significant role of TGF-β2 in these processes, as demonstrated by elevated TGF-β2 levels and active ECM production and remodeling in AGA. This involvement is further supported by a microarray study that showed the upregulation of *TGFB* and the dysregulation of several EMT-related genes in balding scalps compared to non-balding scalps [46].

TWIST1 is a well-established transcriptional regulator of the EMT and fibrosis. It enhances the expression of ECM-related genes, promoting fibrotic remodeling [41,47]. In epithelial cells, TWIST1 represses epithelial markers such as E-cadherin while inducing mesenchymal and ECM-remodeling genes, thereby facilitating the transition of epithelial cells toward a mesenchymal, fibrotic phenotype [48]. In the skin and other fibrotic tissues, TWIST1 has been shown to induce the EMT, activate fibroblasts, and promote collagen deposition, reinforcing its central role in EMT-associated fibrosis [49]. In our study, an upstream regulator analysis identified *TWIST1* as a key transcriptional driver of the EMT and fibrotic remodeling within the progenitor cell niche. *TWIST1* was predicted to activate several downstream effectors, including *FN1* and *TGFB2*, both of which—as previously discussed—are associated with extracellular matrix remodeling and fibrosis. Additionally, *TWIST1* is associated with the upregulation of *CHAD*, a collagen-binding ECM protein that enhances cell–matrix adhesion and has been implicated in pathological ECM remodeling [50,51]. Conversely, *TWIST1* appeared to suppress *CHI3L1*. Although *CHI3L1* is generally linked to pro-fibrotic roles, it can also serve homeostatic and protective functions, particularly during early tissue injury; notably, reduced *CHI3L1* expression may lead to tissue damage and eventual fibrosis [31,52]. According to our study of early-stage AGA, a reduced *CHI3L1* expression may reflect an early tissue injury response. Together, these findings suggest that TWIST1 is not only a marker, but also a potential mediator of EMT-driven progenitor cell loss in a balding scalp.

Moreover, a previous study on AGA showed that *TWIST1* has been identified as being significantly upregulated in DPCs from balding scalps compared to those from non-balding scalps, highlighting its potential involvement in AGA. It is also pinpointed as a gene of functional relevance at one of the AGA risk loci, specifically at 7p21.1 [46]. In the context of prostate cancer, *TWIST1* has been shown to induce the overexpression of the androgen receptor (AR), a critical factor in AGA, thereby potentially increasing the sensitivity of HFs to androgens.

Interestingly, the EMT has been observed in scarring alopecias, such as lichen planopilaris (LPP) and frontal fibrosing alopecia (FFA), which are characterized by irreversible HF loss due to the apoptosis and EMT of epithelial stem cells in the HF bulge [53]. Additionally, fibrosing alopecia in a pattern distribution (FAPD) overlaps with the FFA pathology, but clinically resembles AGA, suggesting a possible common pathophysiology [54]. These conditions may represent different manifestations of a typical disease process involving the EMT. However, despite the presence of the EMT in both scarring alopecias and AGA, variations in the EMT process, such as the degree and chronicity of the EMT, as well as differing inflammation and follicular stem cell involvement, may lead to varying degrees of follicular destruction and fibrosis.

Furthermore, molecular changes within immune cells may significantly affect progenitor cell behavior. Microinflammation, marked by peri-infundibular immune cell infiltration, is believed to induce perifollicular fibrosis [29]. Although not located at the same level within the HF, this immune infiltration can affect progenitor cells through short-range signals or potentially alter the adjacent bulge HFSC [30], the originators of progenitor cells [15], priming them towards a more fibrotic state and fostering an EMT process. Our previous study indicated that CD4^+^ T cells are found in more significant numbers in AGA than in controls and are more likely to trigger a Th2 response [28]. This Th2 response is centrally involved in fibrosis and the EMT, unlike a Th1 response, which is typically associated with anti-fibrotic effects [55,56]. Furthermore, increased cytokines such as *TGFB3* and *FGF6* reveal complex interactions. While *FGF6* is well-known for its role in myogenesis, its role in the EMT and fibrosis remains unclear [57]. However, considering the involvement of FGF signaling in the EMT, *FGF6* could also contribute to these processes [58,59]. Although *TGFB3* generally has anti-fibrotic properties in cutaneous wound healing [60], it can exert a pro-fibrotic effect depending on the tissue context. It has been recently identified as contributing to pathological fibrosis in conditions such as liver fibrosis and systemic sclerosis [61,62]. It also plays a role in inducing the EMT during developmental processes [63]. This intricate network of interactions underscores the impact of immunological changes on transcriptional modifications in progenitor cells in AGA, suggesting a dynamic interplay within the microenvironment that may propel disease progression.

In summary, our study uncovered a previously underrecognized dimension of AGA pathogenesis—molecular and microenvironmental disruptions within the epithelial progenitor cell niche. We demonstrated that this niche is influenced by the EMT and fibrosis. These spatially coordinated alterations suggest the EMT and fibrotic remodeling as promising therapeutic targets beyond the traditional DHT–dermal papilla axis. Although limited by a small sample size, our spatial transcriptomic approach, combined with the protein-level validation of key genes, supports the robustness of the findings. Future studies with larger cohorts are needed to confirm and expand upon these insights. Together, our results provide a valuable spatial framework for understanding early AGA and informing new treatment strategies

## 4. Materials and Methods

### 4.1. Biopsy Collection and Preparation

Under the approval of Thammasat University’s Human Research Ethics Committee (MTU-EC-OO-6-085/64), biopsies were collected from patients with early-stage AGA, specifically those with a Hamilton–Norwood III vertex classification, based on clinical and dermoscopic features. Control donors exhibited no signs of AGA or other hair or scalp disorders. Each subject underwent a 4 mm punch biopsy from the vertex, which was immediately fixed in 10% formalin and then embedded in paraffin. Sequential sections of a 5 μm thickness were prepared for all subsequent analyses.

### 4.2. Slide Preparation for Spatial Transcriptome Profiling

Samples were profiled using the GeoMx DSP (NanoString Technologies, Inc., Seattle, WA, USA). We prepared slides with five tissue sections sourced from three AGA patients and two control donors to optimize the probe hybridization efficiency and mitigate slide-to-slide variability. The sections were distributed randomly across two slides. These sections underwent a standard preparatory process as specified in the GeoMx DSP RNA Slide Preparation Manual (MAN-10101-01) and the GeoMx NGS Library Prep Readout (MAN-10133-03). Briefly, 5-micron-thick, formalin-fixed, paraffin-embedded sections were placed on charged slides, baked to adhere, deparaffinized, and rehydrated. Antigen retrieval was performed using boiling Tris-EDTA (pH of 9), followed by proteinase K treatment. After washing, the slides were hybridized overnight at 37 °C with the Whole Transcriptome Atlas (WTA), which contains probes that are each tagged with a unique photocleavable barcode for sequencing. On the subsequent day, after thoroughly washing and blocking, the slides were incubated with fluorescently tagged antibodies against CD34 (R&D Systems, Minneapolis, MN, USA; clone QBEnd10; catalog number FAB7227T-100UG; dilution 1:100), CD200 (Thermo Fisher Scientific, Waltham, MA, USA; clone OX90; catalog number 14-5200-82; dilution 1:50), and a DNA-binding dye (SYTO™ 83, Thermo Fisher Scientific, Waltham, MA, USA; dilution 1:25) at room temperature for one hour.

The slides were then loaded into the GeoMx DSP and covered in the acquisition buffer. CD34^+^ progenitor cells in the lower portion of the HF were identified as regions of interest (ROIs) using the device interface. Target-specific barcodes were released from each probe using UV light and were collected for library preparation according to NanoString’s guidelines. Sequencing libraries were processed, and raw readouts were aligned to their respective genes via a proprietary barcode-gene matching system provided by NanoString.

### 4.3. Spatial Transcriptome Data Analysis

For the spatial transcriptome data analysis, a definitive list of detectable genes was established by excluding genes below a limit of quantification (LOQ) threshold, which was set at 1% coverage across replicates. The LOQ was determined using the negative controls’ geometric mean and geometric standard deviation within the dataset. After this, each gene’s raw read counts were normalized to the third quartile. We assessed differential gene expression via an unpaired, heteroscedastic t-test on the log2-transformed normalized data. We compared PG-A with PG-C. The threshold for significance in differential gene expression was established at a *p*-value of 0.05 and a log2 fold change of 1.2.

### 4.4. Overrepresentation Analysis

In the overrepresentation analysis, genes showing differential expression were subjected to a Gene Ontology enrichment analysis using gProfiler (https://biit.cs.ut.ee/gprofiler/gost (accessed on 7 July 2022)). Those genes exhibiting a p-value of less than 0.05 and a log2 fold change of 1.2 or greater were evaluated for their enrichment in biological processes, Reactome pathways, and cellular components. Enrichment terms were considered significant if they had a false discovery rate (FDR) of less than 0.05.

### 4.5. Protein–Protein Interaction Network Construction and Analysis

The construction and analysis of predicted functional and physical protein–protein interaction networks were performed using the STRING app version 2.0.1 in Cytoscape version 3.9.1 [64]. Lists of DEGs were imported, and their interactions were evaluated using a confidence cutoff of 0.4. The MCC algorithm within a co-expression network context was identified as the most effective for detecting hub nodes [65]. CytoHubba version 1.5.1, a plugin for Cytoscape, was used to calculate the MCC for each node. For this study, genes ranking in the top 10 MCC values were designated as hub genes. CytoHubba’s parameters included the top ten nodes ranked by MCC values, display options for the first-stage nodes, the identification of the shortest pathway, and the visualization of expanded subnetworks.

### 4.6. Gene Set Enrichment Analysis

A GSEA was conducted using the GSEA platform, version 4.2.2. The data, consisting of gene expression profiles showing the PG-A variation relative to PG-C, were loaded into the GSEA platform. The analysis utilized the Hallmark and Reactome gene sets from the Molecular Signatures Database, running the GSEA with the standard settings. Typically, a GSEA employs a FDR of 0.25 as the threshold for statistical significance [66]. However, this study adopted a more stringent FDR of 0.1 to identify significant enrichment within gene sets while minimizing false positives. The gene sets from PG-A and PG-C that exhibited notable enrichment were identified based on their normalized enrichment score (NES) and FDR. When compared with PG-C, the ggplot2 package in R was utilized to create visual representations of the results, highlighting the dysfunctional pathways in PG-A.

### 4.7. Immunohistochemistry and Digital Image Analysis

Sections (5 μm) from formalin-fixed, paraffin-embedded (FFPE) specimens were prepared and subjected to an immunohistochemical analysis. The sections were incubated with one of three antibodies: mouse monoclonal anti-fibronectin (catalog #14-9869-82), mouse monoclonal anti-TGF beta-2 (catalog #MA5-37505), or sheep polyclonal anti-TWIST1 (catalog #PA5-47824), all obtained from Invitrogen and used at a dilution of 1:100.

The slides were digitized with MoticEasyScan from Motic Digital Pathology. The subsequent image processing and analysis were conducted in QuPath software version 0.1.2, a digital bioimage analysis software from Queen’s University Belfast [67]. This study focused on the sub-bulge and supra-bulbar areas within the lower segment of the HFs. Here, progenitor cells were identified as basal keratinocytes located in these specific regions. These cells were manually annotated to define the ROIs, specifically the PG-A and PG-C regions, for a further detailed analysis.

This analysis included seven patients with AGA and five control donors. In this analysis, 4–5 microscopic fields were obtained from each participant’s section, resulting in 20–35 analyzed fields per group. A microscopic image from each sample was utilized to calibrate the QuPath software, setting clear distinctions between positively stained areas (“Positive”) and the rest (“Negative”) for the purpose of training pixel classifiers [68]. This training was essential for the pixel classifier algorithm to accurately segregate positively stained areas from the negative within each ROI, normalizing the positively stained area to the total area.

Statistical evaluations to discern significant differences between PG-A and PG-C were performed using Python (version 3.11.4), employing the Mann–Whitney U test from the SciPy library (version 1.11.0). Data visualization, including the generation of plots, was carried out using Matplotlib (version 3.8.4).

## 5. Conclusions

**Figure 6:**
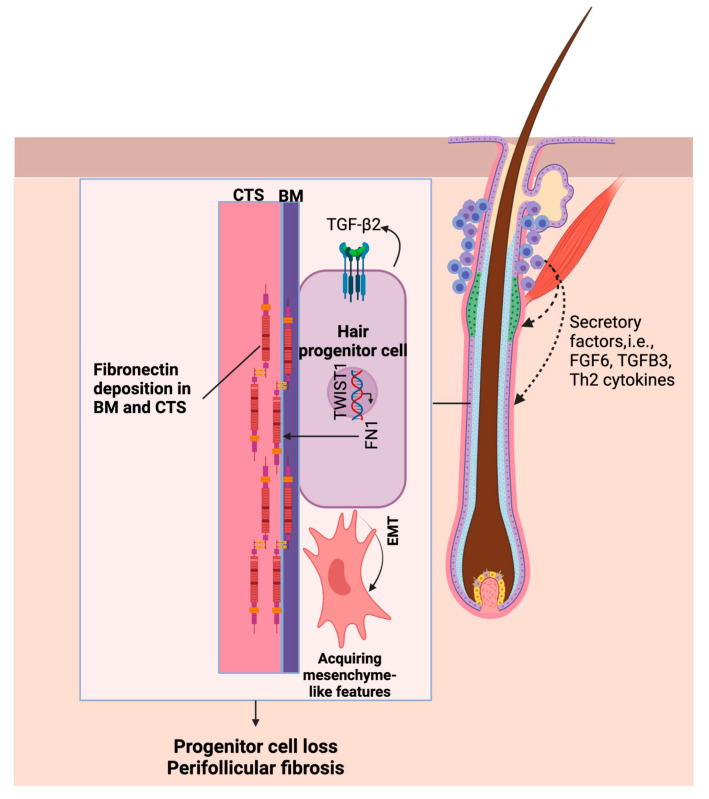
Molecular interactions in AGA, focusing on the roles of TGF-β2, TWIST1, and FN1 within hair follicle (HF) progenitor cells. Autocrine signaling by TGF-β2 activates TWIST1, which plays a pivotal role in regulating genes linked to the epithelial–mesenchymal transition (EMT) and fibrosis. The activation of TWIST1 leads to the increased transcription of FN1, which is crucial for the organization of the extracellular matrix (ECM), resulting in the deposition of fibronectin in the basement membrane (BM) and connective tissue sheath (CTS). These molecular processes create a microenvironment that promotes the EMT, potentially leading to progenitor cell loss and enhancing perifollicular fibrosis, thereby elucidating the key pathophysiological features of AGA. Additionally, signals from immune cells infiltrating around the upper part of the HF may contribute to these processes.

In conclusion, our study identified the loss of progenitor cells, perifollicular fibrosis, and the epithelial-to-mesenchymal transition (EMT) as key contributors to the pathogenesis of AGA (Figure 6). Spatial transcriptomics enabled high-resolution mapping of molecular changes within the HF, revealing potential targets for therapeutic intervention. These findings provide a framework for future studies to dissect upstream regulators and assess clinical applications. More broadly, our results highlight the value of spatially resolved transcriptomic approaches in advancing dermatological research.

## Data Availability

The data generated and analyzed during this study have been deposited in the Gene Expression Omnibus (GEO; http://www.ncbi.nlm.nih.gov/geo) repository and are publicly available under accession number GSE282856.

## Supplementary Materials

The following supporting information can be downloaded at: https://www.mdpi.com/article/10.3390/ijms26125792/s1.
