# Supplementary material for: Progenitor Cell Dynamics in Androgenetic Alopecia: Insights from Spatially Resolved Transcriptomics
Source: Int J Mol Sci. 2025 Jun 17;26(12):5792. doi: 10.3390/ijms26125792 (PMC12193697; doi:10.3390/ijms26125792)
Supplement: Supplementary file 1 [file ijms-26-05792-s001.zip › ijms-3679400-supplementary.pdf]

Supplementary Material

Supplementary Figures and Tables

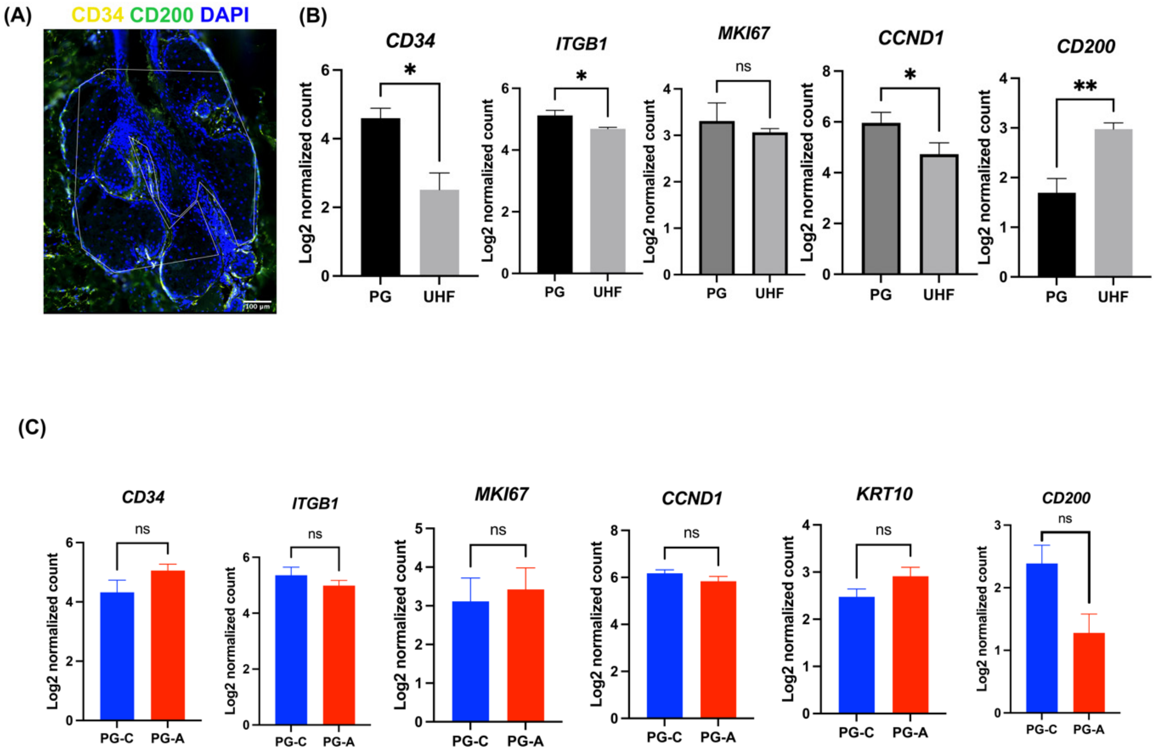

Supplementary Figure S1. Determination of the purity of ROIs. (a) Representative images of the other ROIs (UHF) selected for comparison. (b) Bar graph showing mean Log2 normalized count of the genes from PG vs UHF. (c) Comparison of the level of the genes between PG-A and PG-C. Significance: ns = not significant, **p* < 0.05 and ***p* < 0.01.UHF, upper hair follicle.

Supplementary Table S1. Patient demographic data.

ID	Age	Hamilton-Norwood classification	AGA in first degree relatives	Underlying disease	History of noticeable scalp dermatitis	History of other hair loss disorders	History of using treatments for AGA
**CNT1***	30	I (Normal)	No	none	none	none	none
**CNT2***	28	I (Normal)	No	none	none	none	none
**CNT3**	32	I (Normal)	No	none	none	none	none
**CNT4**	28	I (Normal)	No	none	none	none	none
**AGA1***	27	III vertex	Yes	none	none	none	none
**AGA2***	27	III vertex	Yes	none	none	none	none
**AGA3***	31	III vertex	Yes	none	none	none	none
**AGA4**	29	III vertex	Yes	none	none	none	none
**AGA5**	30	III vertex	Yes	none	none	none	none
**AGA6**	41	III vertex	Yes	none	none	none	none

Patients in this study. Asterisks indicate patients whose tissues were subjected to spatial transcriptome profiling. The tissues of all the patients were used for immunohistochemistry analysis. CNT, control donor; AGA, patient with AGA.

Supplementary Table S2. DEGs with corresponding log2FC and p-value.

Gene symbol	Log2	Pvalue	Regulation
MX1	-2.6684863	0.01829992	Down-regulated
PNO1	-2.2815771	0.00049214	Down-regulated
CPA4	-2.0257565	0.00475303	Down-regulated
SUPT7L	-1.8893165	4.5247E-05	Down-regulated
FAM83F	-1.8524657	0.00274176	Down-regulated
SCNN1B	-1.784675	0.01803252	Down-regulated
DCANP1	-1.7791413	0.02675922	Down-regulated
SIRPA	-1.7041079	0.00868287	Down-regulated
SERINC2	-1.6949984	0.01494697	Down-regulated
KRTAP5-8	-1.68663	0.03791227	Down-regulated
PIGH	-1.6850468	0.02164523	Down-regulated
C22orf39	-1.6797605	0.0272107	Down-regulated
OSGIN1	-1.6617522	0.00621877	Down-regulated
UHRF1	-1.6470188	0.01188949	Down-regulated
CCDC13	-1.6452039	0.0344457	Down-regulated
WDR44	-1.6395082	0.00310701	Down-regulated
TNIP2	-1.6276953	0.04849992	Down-regulated
KCNK4	-1.6258223	0.00704425	Down-regulated

HELZ2	-1.6027757	0.02482172	Down-regulated
AIF1	-1.6020972	0.03059629	Down-regulated
PINLYP	-1.596118	0.00149441	Down-regulated
MTMR1	-1.5661678	0.01089015	Down-regulated
PLAGL2	-1.5606868	0.01522766	Down-regulated
SLC30A1	-1.5596676	0.0070679	Down-regulated
PYGL	-1.5587744	0.01083429	Down-regulated
NIPAL2	-1.5409502	0.00872368	Down-regulated
ILVBL	-1.5398061	0.00085689	Down-regulated
RESP18	-1.5385606	0.02288459	Down-regulated
TRIM38	-1.5346344	0.03200916	Down-regulated
ALDH3B2	-1.526422	0.00104101	Down-regulated
TEX22	-1.5178251	0.0092417	Down-regulated
ING2	-1.5168789	0.03273339	Down-regulated
GADD45A	-1.5099277	0.03082001	Down-regulated
RNASEH2A	-1.5085706	0.01020939	Down-regulated
GRHL1	-1.5011171	0.00104389	Down-regulated
ORC1	-1.491252	0.02299784	Down-regulated
OR10G3	-1.4871992	0.0044744	Down-regulated
CES2	-1.4820448	0.01686004	Down-regulated
IL33	-1.4769065	0.00249464	Down-regulated
NSD2	-1.4767731	0.01511311	Down-regulated
SEMA4A	-1.4752531	0.02767033	Down-regulated
CNTLN	-1.4708705	0.00453098	Down-regulated
THUMPD2	-1.4675032	0.00034088	Down-regulated
TSPAN2	-1.4623793	0.01146657	Down-regulated
SLC34A1	-1.4532721	0.02046198	Down-regulated
FLOT2	-1.4354056	0.03557226	Down-regulated
LGALS3BP	-1.4305354	0.03501616	Down-regulated
NSUN4	-1.4239577	0.03658449	Down-regulated
NTRK2	-1.4211228	0.00092819	Down-regulated
PLSCR1	-1.4153526	0.00442089	Down-regulated
PARP9	-1.413179	0.0029447	Down-regulated
SLC36A1	-1.4027486	0.03338287	Down-regulated
LPIN1	-1.4009045	0.02162076	Down-regulated
MESP1	-1.39718	0.00039625	Down-regulated
SIGLEC15	-1.3904696	0.0053681	Down-regulated
TMEM79	-1.3855932	0.00277879	Down-regulated

CHI3L1	-1.3760835	0.0040907	Down-regulated
ENC1	-1.3714891	0.02253939	Down-regulated
SF3B3	-1.3710566	0.0099709	Down-regulated
NCBP2L	-1.370094	0.03964029	Down-regulated
NFKBIL1	-1.3654195	0.03476558	Down-regulated
ANO8	-1.3623036	0.04431195	Down-regulated
CCDC87	-1.362133	0.00921857	Down-regulated
CTSD	-1.360668	0.04486605	Down-regulated
AGPAT1	-1.357316	0.01582026	Down-regulated
TBC1D2	-1.3557079	0.02793685	Down-regulated
CEBPD	-1.3491533	0.03527269	Down-regulated
SMARCD2	-1.3490532	0.00085658	Down-regulated
SDC1	-1.3485674	0.01532406	Down-regulated
ATP5MC2	-1.3464603	0.04423304	Down-regulated
C5orf63	-1.3423102	0.04949024	Down-regulated
TMEM200A	-1.3388405	0.03997499	Down-regulated
MRRF	-1.3378188	0.01535588	Down-regulated
APLP1	-1.3362796	0.00964429	Down-regulated
LOXL1	-1.3360042	0.00157501	Down-regulated
SLC44A1	-1.3341252	0.00197716	Down-regulated
KRT9	-1.3324154	0.00320235	Down-regulated
CASP10	-1.326128	0.00283842	Down-regulated
GAB1	-1.326084	0.02613504	Down-regulated
VHL	-1.3179168	0.02149631	Down-regulated
CRISP2	-1.3129958	0.00167025	Down-regulated
NMS	-1.3108915	0.0019922	Down-regulated
RTCA	-1.3103609	0.00827259	Down-regulated
PIP4K2C	-1.3102158	0.01009621	Down-regulated
SRF	-1.3093167	0.00677549	Down-regulated
SPDYA	-1.3053484	0.00533172	Down-regulated
FAM83B	-1.3015408	0.01899416	Down-regulated
RNF4	-1.2997316	0.02394928	Down-regulated
APH1B	-1.2973348	0.0045044	Down-regulated
EDEM2	-1.2940538	0.00485091	Down-regulated
CD82	-1.2929972	0.02083321	Down-regulated
TAF5L	-1.2919843	0.00871626	Down-regulated
SACS	-1.2877224	0.01446042	Down-regulated
MPST	-1.2849678	0.0344411	Down-regulated

SCARB1	-1.2816202	0.00818642	Down-regulated
C6orf58	-1.2815771	0.04339461	Down-regulated
S1PR3	-1.2791255	0.00724358	Down-regulated
TBC1D7	-1.2775982	0.04389465	Down-regulated
GRIK3	-1.2771742	0.03028607	Down-regulated
IVL	-1.2735298	0.00535728	Down-regulated
APOC3	-1.2721738	0.01089831	Down-regulated
PDCD4	-1.2710468	0.00393564	Down-regulated
TKT	-1.2687772	0.00153869	Down-regulated
KIAA1549L	-1.2632971	0.00623236	Down-regulated
MARCHF6	-1.2611152	0.0123829	Down-regulated
PNLDC1	-1.2532721	0.04447959	Down-regulated
ANKRD9	-1.2528844	0.009383	Down-regulated
MINDY2	-1.2508205	0.03744691	Down-regulated
MXD1	-1.250352	5.9284E-05	Down-regulated
IKBKE	-1.2487068	0.04865984	Down-regulated
PARP12	-1.2466123	0.00360465	Down-regulated
SERINC5	-1.2422998	0.00458878	Down-regulated
GPSM2	-1.2417732	0.00802162	Down-regulated
TMEM41B	-1.2410365	0.00726986	Down-regulated
ARHGAP19	-1.2390236	0.01969659	Down-regulated
ATP1A1	-1.2368409	0.00542334	Down-regulated
OTULINL	-1.2367607	0.03676065	Down-regulated
PNPLA4	-1.2328344	0.01900207	Down-regulated
MYLK	-1.2322406	0.01945424	Down-regulated
FLRT3	-1.230295	0.0145261	Down-regulated
GNA15	-1.2277574	0.00744621	Down-regulated
SLC22A3	-1.2256008	0.03540483	Down-regulated
KCNJ9	-1.2237654	0.03175349	Down-regulated
KDM4E	-1.2226544	0.02745065	Down-regulated
PRKAR2B	-1.2149105	0.02827684	Down-regulated
PTGFRN	-1.2084039	0.04656997	Down-regulated
HACD2	-1.2079588	0.01228847	Down-regulated
SLC22A23	-1.2042078	0.00766057	Down-regulated
NHLRC1	-1.2035821	0.00345234	Down-regulated
TJAP1	-1.2029463	0.03186468	Down-regulated
IPPK	-1.2013011	0.03216398	Down-regulated
GALK2	1.20397894	0.00245493	Up-reguated

SEPTIN11	1.20936644	0.00969552	Up-reguated
FSTL1	1.21111675	0.0352294	Up-reguated
BLOC1S2	1.2169098	0.012729	Up-reguated
DENND2B	1.21805035	0.02090991	Up-reguated
LNPEP	1.23306279	0.01734434	Up-reguated
SCARF2	1.23440926	0.02158133	Up-reguated
LRRC37B	1.24418286	0.03169246	Up-reguated
PCSK9	1.24848713	0.02505224	Up-reguated
PHF2	1.25594997	0.03125668	Up-reguated
TMSB4X	1.2584862	0.02367581	Up-reguated
BTD	1.26099217	0.0302442	Up-reguated
MDFIC2	1.27505196	0.04983278	Up-reguated
HAGHL	1.29222764	0.03197015	Up-reguated
PITX1	1.29408027	0.02302926	Up-reguated
LCE1F	1.29450869	0.00840031	Up-reguated
SHOC1	1.29952119	0.0201905	Up-reguated
CRNKL1	1.30856848	0.01167166	Up-reguated
ITPRIPL2	1.31121034	0.00865965	Up-reguated
HTRA1	1.31704438	0.00516365	Up-reguated
REEP5	1.31706718	0.0019763	Up-reguated
SRFBP1	1.32087452	0.0142074	Up-reguated
TBC1D30	1.32993989	0.03061352	Up-reguated
BTBD19	1.34984702	0.03099167	Up-reguated
CCDC77	1.36034051	0.02445637	Up-reguated
OR4E2	1.37751619	0.03792046	Up-reguated
IRF4	1.38611307	0.01810078	Up-reguated
ZNF566	1.39157598	0.03013758	Up-reguated
TDRP	1.39255369	0.0046696	Up-reguated
NT5C	1.39874113	0.0012241	Up-reguated
SPTBN1	1.4180296	0.00199486	Up-reguated
AXDND1	1.43012307	0.03153271	Up-reguated
CMIP	1.47185202	0.01963081	Up-reguated
AGTPBP1	1.47880371	0.02723502	Up-reguated
ULK4	1.48044891	0.00472809	Up-reguated
GRAPL	1.48089931	0.04576266	Up-reguated
DAZAP2	1.48186126	0.02222355	Up-reguated
YLPM1	1.49341472	0.01374119	Up-reguated
CREM	1.49724014	0.04965019	Up-reguated

DCTN1	1.50583702	0.01581244	Up-reguated
TIMP2	1.50643684	0.02987508	Up-reguated
BMT2	1.51094644	0.01139214	Up-reguated
TTYH2	1.51129993	0.01333523	Up-reguated
MAP1LC3A	1.5220094	0.01682379	Up-reguated
SERTAD4	1.57052053	0.04682859	Up-reguated
CEP20	1.60793489	0.00668684	Up-reguated
COL17A1	1.61028775	0.00244425	Up-reguated
SERPINE2	1.61409355	0.0018585	Up-reguated
POSTN	1.62312796	0.03396207	Up-reguated
ZNF578	1.62408918	0.04407875	Up-reguated
GTF2H3	1.66683952	0.01426473	Up-reguated
SLC6A6	1.68161281	0.00410751	Up-reguated
SNX10	1.69678974	0.0136389	Up-reguated
GAB2	1.71322748	0.01620121	Up-reguated
IRX6	1.76390681	0.02375911	Up-reguated
TGFB2	1.79270872	0.00551668	Up-reguated
SFXN2	1.83057348	0.04315346	Up-reguated
GREM1	1.84253766	0.01199762	Up-reguated
CLDN23	1.84575568	0.01225102	Up-reguated
THBS1	1.84657006	0.00388299	Up-reguated
COL3A1	1.92120392	0.02431897	Up-reguated
FN1	1.94780906	0.00818516	Up-reguated
OR2B11	1.98931301	0.00130225	Up-reguated
CHAD	2.05277974	0.02131705	Up-reguated
KRT15	2.05859086	0.03116463	Up-reguated
PLXNC1	2.26182702	0.00389172	Up-reguated

Supplementary Table S3. Gene set enrichment analysis (GSEA) of the ranked list of genes from the expression dataset of PG-A compared with PG-C based on Reactome and Hallmark gene sets with FDR < 0.1.

NAME	SIZE	NES	FDR
REACTOME_MET_ACTIVATES_PTK2_SIGNALING	26	2.164398	0.002350723
REACTOME_COLLAGEN_CHAIN_TRIMERIZATION	33	2.2312086	0.002858369
REACTOME_ASSEMBLY_OF_COLLAGEN_FIBRILS_AND_OTHER_MULTIMERIC_STRUCTURES	50	2.1880488	0.002998773
REACTOME_COLLAGEN_BIOSYNTHESIS_AND_MODIFYING_ENZYMES	49	1.9632152	0.02809512
HALLMARK_EPITHELIAL_MESENCHYMAL_TRANSITION	169	1.9033785	0.032992702
REACTOME_MET_PROMOTES_CELL_MOTILITY	37	1.9104663	0.03609697

REACTOME_COLLAGEN_FORMATION	68	1.9132955	0.041655328
REACTOME_MOLECULES_ASSOCIATED_WITH_ELASTIC_FIBRES	30	1.7638994	0.0838481
REACTOME_COLLAGEN_DEGRADATION	47	1.7739202	0.08493043
REACTOME_ECM_PROTEOGLYCANS	56	1.7831316	0.086258724
REACTOME_DEGRADATION_OF_THE_EXTRACELLULAR_MATRIX	104	1.7916192	0.08843947

NES, normalized enrichment score; FDR, false discovery rate.

Supplementary Table S4. Over-represented gene ontology biological process (GOBP) and Reactome (REAC) terms enriched by up- and down-regulated genes

source	term_name	term_id	FDR	genes	direction
GOBP	regulation of bone trabecula formation	GO:1900154	0.00420074040480344	*CHAD,GREM1*	up
GOBP	negative regulation of bone trabecula formation	GO:1900155	0.00420074040480344	*CHAD,GREM1*	up
REAC	Non-integrin membrane-ECM interactions	REAC:R-HSA-3000171	0.0323002311129251	*FN1,COL3A1,THBS1*	up
REAC	Platelet degranulation	REAC:R-HSA-114608	0.0323002311129251	*FN1,THBS1,TGFB2,TMSB4X*	up
REAC	Response to elevated platelet cytosolic Ca2+	REAC:R-HSA-76005	0.0323002311129251	*FN1,THBS1,TGFB2,TMSB4X*	up
REAC	Degradation of the extracellular matrix	REAC:R-HSA-1474228	0.0058321395233339	*FN1,COL3A1,COL17A1,TIMP2,HTRA1*	up
REAC	Extracellular matrix organization	REAC:R-HSA-1474244	0.0058321395233339	*FN1,COL3A1,THBS1,TGFB2,COL17A1,TIMP2,HTRA1*	up
REAC	Syndecan interactions	REAC:R-HSA-3000170	0.0058321395233339	*FN1,COL3A1,THBS1*	up
REAC	Signaling by FLT3 fusion proteins	REAC:R-HSA-9703465	0.0448822845801664	*GAB2,SPTBN1*	up
GOBP	transforming growth factor beta production	GO:0071604	0.00420074040480344	*FN1,COL3A1,THBS1,TGFB2*	up

GOBP	transmembrane receptor protein serine/threonine kinase signaling pathway	GO:0007178	0.00420074040480344	*COL3A1,THBS1,GREM1,TGFB2,SPTBN1,HTRA1,LNPEP,FSTL1*	up
GOBP	peptide cross-linking	GO:0018149	0.0413518718245415	*FN1,COL3A1,THBS1*	up
GOBP	cell-substrate junction assembly	GO:0007044	0.0413518718245415	*FN1,THBS1,GREM1,COL17A1*	up
GOBP	negative regulation of plasminogen activation	GO:0010757	0.0413518718245415	*THBS1,SERPINE2*	up
GOBP	regulation of transforming growth factor beta production	GO:0071634	0.0413518718245415	*FN1,THBS1,TGFB2*	up
GOBP	cell-substrate junction organization	GO:0150115	0.0413518718245415	*FN1,THBS1,GREM1,COL17A1*	up
GOBP	organophosphate biosynthetic process	GO:0090407	0.00511589604295799	*IPPK,HACD2,SERINC5,TKT,PIP4K2C,SLC44A1,ATP5MC2,AGPAT1,LPIN1,PARP9,PLSCR1,PYGL,MTMR1,PIGH*	down
GOBP	lipid biosynthetic process	GO:0008610	0.0486790397398182	*HACD2,ATP1A1,SERINC5,APOC3,SCARB1,PIP4K2C,SLC44A1,AGPAT1,LPIN1,PLSCR1,ALDH3B2,MTMR1,PIGH*	down
GOBP	phospholipid biosynthetic process	GO:0008654	0.0424367580969031	*SERINC5,PIP4K2C,SLC44A1,AGPAT1,LPIN1,PLSCR1,MTMR1,PIGH*	down
GOBP	glycerolipid metabolic process	GO:0046486	0.00511589604295799	*PNPLA4,SERINC5,APOC3,SCARB1,PIP4K2C,SLC44A1,AGPAT1,LPIN1,PLSCR1,MTMR1,PIGH,SERINC2*	down
GOBP	phospholipid metabolic process	GO:0006644	0.0375790669488242	*SERINC5,SCARB1,PIP4K2C,SLC44A1,AGPAT1,LPIN1,PLSCR1,MTMR1,PIGH,SERINC2*	down
GOBP	glycerolipid biosynthetic process	GO:0045017	0.0375790669488242	*SCARB1,PIP4K2C,SLC44A1,AGPAT1,LPIN1,PLSCR1,MTMR1,PIGH*	down
GOBP	cellular lipid metabolic process	GO:0044255	0.0375790669488242	*HACD2,PRKAR2B,PNPLA4,SERINC5,APOC3,SCARB1,PIP4K2C,SLC44A1,AGPAT1,LPIN1,PLSCR1,CES2,ALDH3B2,ILVBL,MTMR1,PIGH,SERINC2*	down

GOBP	lipid metabolic process	GO:0006629	0.0375790669488242	*HACD2,PRKAR2B,PNPLA4,ATP1A1,SERINC5,APOC3,SCARB1,PIP4K2C,SLC44A1,AGPAT1,LPIN1,PLSCR1,CES2,ALDH3B2,ILVBL,PLAGL2,MTMR1,PIGH,SERINC2,SCNN1B*	down
GOBP	glycerophospholipid metabolic process	GO:0006650	0.0110461272695089	*SERINC5,SCARB1,PIP4K2C,SLC44A1,AGPAT1,LPIN1,PLSCR1,MTMR1,PIGH,SERINC2*	down
GOBP	organophosphate metabolic process	GO:0019637	0.0375790669488242	*IPPK,HACD2,SERINC5,TKT,SCARB1,PIP4K2C,SLC44A1,ATP5MC2,AGPAT1,LPIN1,PARP9,PLSCR1,PYGL,MTMR1,PIGH, SERINC2*	down

GOBP, gene ontology biological process; REAC, Reactome pathway; FDR, false discovery rate; genes; 'up', from up-regulated gene set; 'down', from down-regulated gene set.

Supplementary Table S5. Over-represented gene ontology cellular component (GOCC) terms enriched by up- and down-regulated genes

source	term_name	term_id	FDR	genes	direction
GOCC	external encapsulating structure	GO:0030312	2.73370041930039e-05	*CHAD,FN1,COL3A1,THBS1, GREM1,TGFB2,POSTN,SERPINE2,COL17A1,TIMP2,HTRA1*	up
GOCC	endoplasmic reticulum lumen	GO:0005788	0.00814218967192879	*FN1,COL3A1,THBS1,COL17A1,PCSK9,FSTL1*	up
GOCC	extracellular matrix	GO:0031012	2.73370041930039e-05	*CHAD,FN1,COL3A1,THBS1, GREM1,TGFB2,POSTN,SERPINE2,COL17A1,TIMP2,HTRA1*	up
GOCC	secretory granule lumen	GO:0034774	0.0485862468088031	*FN1,THBS1,TGFB2,TIMP2,TMSB4X*	up
GOCC	cytoplasmic vesicle lumen	GO:0060205	0.0485862468088031	*FN1,THBS1,TGFB2,TIMP2,TMSB4X*	up
GOCC	extrinsic component of external side of plasma membrane	GO:0031232	0.00914393774827258	*SERPINE2,PCSK9*	up
GOCC	vesicle lumen	GO:0031983	0.0485862468088031	*FN1,THBS1,TGFB2,TIMP2,TMSB4X*	up
GOCC	fibrinogen complex	GO:0005577	0.00814218967192879	*FN1,THBS1*	up

GOCC	platelet alpha granule lumen	GO:0031093	0.00177328502691259	*FN1,THBS1,TGFB2,TMSB4X*	up
GOCC	platelet alpha granule	GO:0031091	0.000325758934212826	*FN1,THBS1,TGFB2,SERPINE2,TMSB4X*	up
GOCC	collagen-containing extracellular matrix	GO:0062023	2.73370041930039e-05	*FN1,COL3A1,THBS1,GREM1,TGFB2,POSTN,SERPINE2,COL17A1,TIMP2,HTRA1*	up
GOCC	nucleoplasm	GO:0005654	0.026310468782266	*IPPK,IKBKE,MXD1,MINDY2,TKT,IVL,TAF5L,RNF4,SPDYA,SRF,PIP4K2C,RTCA,VHL,SLC44A1,APLP1,SMARCD2,CEBPD,TBC1D2,NFKBIL1,SF3B3,ENC1,LPIN1,PARP9,PLSCR1,SLC34A1,TSPAN2,CNTLN,NSD2,IL33,ORC1,GRHL1,RNASEH2A,GADD45A,ING2,TRIM38,HELZ2,TNIP2,CCDC13,UHRF1,DCANP1,SUPT7L,PNO1*	down
GOCC	endoplasmic reticulum	GO:0005783	0.026310468782266	*NHLRC1,HACD2,PTGFRN,FLRT3,OTULINL,ATP1A1,TMEM41B,PNLDC1,MARCHF6,TKT,EDEM2,APH1B,PIP4K2C,VHL,AGPAT1,ANO8,CHI3L1,LPIN1,SLC36A1,CES2,RESP18,ILVBL,SLC30A1,PIGH,MX1*	down
GOCC	cytoplasm	GO:0005737	0.026310468782266	*IPPK,TJAP1,NHLRC1,HACD2,PTGFRN,PRKAR2B,FLRT3,MYLK,PNPLA4,OTULINL,ATP1A1,ARHGAP19,TMEM41B,GPSM2,SERINC5,IKBKE,MXD1,ANKRD9,PNLDC1,MARCHF6,TKT,PDCD4,APOC3,IVL,GRIK3,TBC1D7,S1PR3,SCARB1,MPST,SACS,TAF5L,EDEM2,APH1B,RNF4,FAM83B,SRF,PIP4K2C,VHL,GAB1,CASP10,KRT9,SLC44A1,LOXL1,APLP1,MRRF,ATP5MC2,SDC1,TBC1D2,AGPAT1,CTSD,ANO8,NFKBIL1,ENC1,CHI3L1,TMEM79,LPIN1,SLC36A1,PARP9,PLSCR1,NTRK2,NSUN4,LGALS3BP,FLOT2,SLC34A1,CNTLN,NSD2,IL33,CES2,ORC*	down

				1,RNASEH2A,GADD45A,TEX22,ALDH3B2,TRIM38,RESP18,ILVBL,PYGL,SLC30A1,MTMR1,AIF1,HELZ2,TNIP2,WDR44,CCDC13,PIGH,KRTAP5-8,SIRPA,DCANP1,SCNN1B,PNO1,MX1	
GOCC	extracellular membrane-bounded organelle	GO:0065010	0.048890464654757	*PRKAR2B,ATP1A1,SERINC5,TKT,APOC3,IVL,C6ORF58,SCARB1,MPST,CD82,PIP4K2C,KRT9,SLC44A1,SDC1,CTSD,CHI3L1,PLSCR1,LGALS3BP,FLOT2,CNTLN,PYGL,SERINC2,SIRPA,SCNN1B*	down
GOCC	extracellular exosome	GO:0070062	0.048890464654757	*PRKAR2B,ATP1A1,SERINC5,TKT,APOC3,IVL,C6ORF58,SCARB1,MPST,CD82,PIP4K2C,KRT9,SLC44A1,SDC1,CTSD,CHI3L1,PLSCR1,LGALS3BP,FLOT2,CNTLN,PYGL,SERINC2,SIRPA,SCNN1B*	down
GOCC	extracellular vesicle	GO:1903561	0.048890464654757	*PRKAR2B,ATP1A1,SERINC5,TKT,APOC3,IVL,C6ORF58,SCARB1,MPST,CD82,PIP4K2C,KRT9,SLC44A1,SDC1,CTSD,CHI3L1,PLSCR1,LGALS3BP,FLOT2,CNTLN,PYGL,SERINC2,SIRPA,SCNN1B*	down
GOCC	extracellular organelle	GO:0043230	0.048890464654757	*PRKAR2B,ATP1A1,SERINC5,TKT,APOC3,IVL,C6ORF58,SCARB1,MPST,CD82,PIP4K2C,KRT9,SLC44A1,SDC1,CTSD,CHI3L1,PLSCR1,LGALS3BP,FLOT2,CNTLN,PYGL,SERINC2,SIRPA,SCNN1B*	down
GOCC	endomembrane system	GO:0012505	0.048890464654757	*TJAP1,NHLRC1,HACD2,PTGFRN,FLRT3,OTULINL,ATP1A1,TMEM41B,SERINC5,PNLDC1,MARCHF6,TKT,APOC3,EDEM2,APH1B,PIP4K2C,VHL,LOXL1,APLP1,SDC1,AGPAT1,CTSD,ANO8,CHI3L1,TMEM79,LPIN1,SLC36A1,PLSCR1,NTRK2,LGALS3BP,FLOT2,SLC34A1,IL33,CES2,TEX22,RESP18,ILVBL,PYGL,SLC30A1,WDR44,PIGH,SIRPA,MX1*	down

GOCC	perinuclear region of cytoplasm	GO:0048471	0.048890464654757	*NHLRC1,PRKAR2B,SERINC5,APLP1,CHI3L1,PLSCR1,NTRK2,FLOT2,SLC34A1,AIF1,WDR44,MX1*	down

GOCC, gene ontology cellular component; FDR, false discovery rate; genes; 'up', from up-regulated gene set; 'down', from down-regulated gene set.

Supplementary Table S6. Top 10 over-represented Kyoto encyclopedia of genes and genomes (KEGG) pathways and gene ontology biological process (GOBP) terms enriched by the combined set of upregulated genes from the progenitor and immune datasets.

**Source**	**Term name**	**P-value**	**Genes**
KEGG_2021_Human	Amoebiasis	0.0002445895721631478	*TGFB2;COL3A1;TGFB3;IL1R2;FN1;RAB7A*
KEGG_2021_Human	Malaria	0.0008744834203776795	*CD40;TGFB2;TGFB3;THBS1*
KEGG_2021_Human	TGF-beta signaling pathway	0.0012809683567569	*GREM1;TGFB2;TGFB3;RGMB;THBS1*
KEGG_2021_Human	Th17 cell differentiation	0.0022701426200293	*CD4;IRF4;HLA-DRA;IL1RAP;IL6R*
KEGG_2021_Human	Human T-cell leukemia virus 1 infection	0.0028275099605159	*TGFB2;CD4;CD40;TGFB3;IL1R2;CANX;HLA-DRA*
KEGG_2021_Human	Antigen processing and presentation	0.0045021592240989	*CD4;CANX;HLA-DRA;TAPBP*
KEGG_2021_Human	Hematopoietic cell lineage	0.0103528159139704	*CD4;IL1R2;HLA-DRA;IL6R*
KEGG_2021_Human	AGE-RAGE signaling pathway in diabetic complications	0.010713918369074	*TGFB2;COL3A1;TGFB3;FN1*
KEGG_2021_Human	Cytokine-cytokine receptor interaction	0.01367110459569	*TGFB2;CD4;CD40;TGFB3;IL1R2;IL1RAP;IL6R*
KEGG_2021_Human	Toxoplasmosis	0.0156918279806361	*TGFB2;CD40;TGFB3;HLA-DRA*
GO_Biological_Process_2023	Regulation Of CD4-positive, Alpha-Beta T Cell Differentiation (GO:0043370)	9.6347704698671e-05	*IRF4;HLA-DRA;SH3RF1*
GO_Biological_Process_2023	Response To Progesterone (GO:0032570)	0.0001649123586008043	*TGFB2;TGFB3;THBS1*
GO_Biological_Process_2023	Response To Ketone (GO:1901654)	0.0006290388305487405	*TGFB2;TGFB3;THBS1*

GO_Biological_Process_2023	Regulation Of Cell Motility (GO:2000145)	0.0006662133623225341	*FGF6;SERPINE2;TMSB4X;ENPP2;PLXNC1;FUT3*
GO_Biological_Process_2023	Phagosome Acidification (GO:0090383)	0.000714561460712844	*RAB38;RAB7A*
GO_Biological_Process_2023	Extracellular Matrix Organization (GO:0030198)	0.000810488330713243	*COL17A1;GREM1;TGFB2;POSTN;COL3A1;ADAM15;MMP28*
GO_Biological_Process_2023	Regulation Of Stress-Activated MAPK Cascade (GO:0032872)	0.000942640695045941	*GREM1;TGFB2;ULK4;PPIA*
GO_Biological_Process_2023	Negative Regulation Of Plasminogen Activation (GO:0010757)	0.0010658184844478	*SERPINE2;THBS1*
GO_Biological_Process_2023	Regulation Of Transforming Growth Factor Beta1 Production (GO:0032908)	0.0010658184844478	*THBS1;TSKU*
GO_Biological_Process_2023	Gland Morphogenesis (GO:0022612)	0.0010658184844478	*TGFB2;TGFB3*
